# γδ T Cell in Cerebral Ischemic Stroke: Characteristic, Immunity-Inflammatory Role, and Therapy

**DOI:** 10.3389/fneur.2022.842212

**Published:** 2022-03-31

**Authors:** Li Wang, Chengye Yao, Jiayi Chen, Yangyang Ge, Chenchen Wang, Yu Wang, Fuquan Wang, Yan Sun, Maosha Dai, Yun Lin, Shanglong Yao

**Affiliations:** ^1^Department of Anesthesiology, Union Hospital, Tongji Medical College, Huazhong University of Science and Technology, Wuhan, China; ^2^Department of Neurology, Union Hospital, Tongji Medical College, Huazhong University of Science and Technology, Wuhan, China

**Keywords:** immunity, inflammation, γδ T, cerebral ischemia stroke, therapy

## Abstract

Gamma-delta (γδ) T cells are a small subset of T cells that are reported to have a proinflammatory role in the pathophysiology of cerebral ischemia stroke (CIS). Upon activation by interleukin-1 beta (IL-1β), IL-23 and IL-18, γδ T cells are stimulated to secrete various cytokines, such as IL-17a, IL-21, IL-22, and interferon-gamma (IFN-γ). In addition, they all play a pivotal role in the inflammatory and immune responses in ischemia. Nevertheless, the exact mechanisms responsible for γδ T cell proinflammatory functions remain poorly understood, and more effective therapies targeting at γδ T cells and cytokines they release remain to be explored, particularly in the context of CIS. CIS is the second most common cause of death and the major cause of permanent disability in adults worldwide. In this review, we focus on the neuroinflammatory and immune functions of γδ T cells and related cytokines, intending to understand their roles in CIS, which may be crucial for the development of novel effective clinical applications.

## Introduction

Stroke, a common disease, is the second leading cause of death and the third leading cause of disability in adults worldwide and places an increasing burden on families and communities (1). Stroke can be broadly divided into the ischemic and hemorrhagic stroke, and the former makes up ~87% of all stroke cases (2). Nowadays, clinical treatment of ischemic stroke is limited to interventions that restore blood flow through either pharmacological thrombolysis—tissue plasminogen activator (tPA), the only approved therapeutic agent—or mechanical thrombectomy. However, due to the moderate recanalization rate, limited time window, adverse effects, and some thrombolytic contraindications, only a small number of patients could receive and benefit from the therapies in time (3). Therefore, it is necessary to continue to explore the pathogenic mechanisms contributing to neurologic injury following a stroke to develop alternative therapeutic strategies.

Severe stenosis or occlusion of a cerebral artery, especially the middle cerebral artery, deprives nerve cells, including neurons and glial cells, of nutrition, such as oxygen, glucose, and lipids (4). Ischemia triggers a complex cascade of events that include energy failure (5), ion imbalance, excitotoxicity (6), oxidative stress (7), cell death, activation of microglia and complement system (8–10), and initiate the inflammation and immune responses. The cascades eventually lead to irreversible brain damage (11). After recanalizing successfully, restoring blood flowing to the ischemic brain causes secondary reperfusion injury. Reperfusion results in the production of reactive oxygen species (ROS) and the amplification of inflammation and immune responses, which subsequently cause undue neural death, impairment of the integrity of the blood-brain barrier (BBB), and activation of innate and adaptive immune systems, and eventually lead to brain damage even worse (12–15). In addition, the affected brain tissue also accumulates specific proinflammatory cells, especially neutrophils, macrophages, and T cells; Of which, T lymphocytes are one of the critical factors to the entire pathophysiology process of CIS (16, 17).

T lymphocytes develop from bone marrow-derived precursors and mature in the thymus after a complex developmental sequence associated with differentiation, selection, and proliferation episodes. They exert a central role in the adaptive immune system and are important in the crosstalk between innate and adaptive immune systems (18). T cells recognize antigens through T cell antigen receptor (TCR) composed of two distinct polypeptide chains (19), two possible pairs of which have been identified: TCRα and TCRβ, or TCRγ and TCRδ, defining αβ and γδ T cells, respectively. Certainly, all types of T cells are closely involved in inflammatory events in stroke (20). Notably, an interest in γδ T cells grows rapidly owing to their essential contributions to immunopathology in many diseases including CIS, therefore, γδ T cells are the focus of our review.

## Characteristics of γδ T Cells

γδ T cells are divided according to the type of Vγ and Vδ chain they express at the TCRs. Of note, classification is one of the differences between human and mouse γδ T cells. In human, three main Vδ gene segments, Vδ1, Vδ2, and Vδ3, are most frequently used in the rearrangement of the δ chain, while seven functional Vγ gene segments, Vγ2, Vγ3, Vγ4, Vγ5, Vγ8, Vγ9, and Vγ11 are used for rearrangement of the γ chain (21). In human beings and Non-human primates along with a few other species, Vγ9Vδ2 T cells are the major γδ T cell subset and are the potential therapeutic target in many diseases (22, 23). While in mice γδ T cell subsets are named according to the Vγ chain used, which contains Vγ1, Vγ4, Vγ5, Vγ6, and Vγ7. Thus, the characteristics describing the γδ T cell subsets of one particular species cannot be applied to another species directly because each repertoire is unique (24). Hence, the experimental results obtained from murine studies need further validation before they are applied to clinical practices.

During fetal thymic development, γδ T cells are the first T cells to appear in the thymus. However, the relative proportion of γδ T cells decreases with the emergence and development of αβ T cells. In adult humans, γδ T cells make up 3–10% of T cells in the peripheral blood (25–27). Certain tissues, including lung, skin, thymus, lymph node, spleen, and breast, also find a similar frequency of γδ T cells (26, 28, 29). In comparison, γδ T cells can constitute up to 30% of all T cells in some compartments of the intestinal tract (30). While in adult mice, γδ T cells constitute 1–4% of total T cells in the blood, lymph node, liver, and spleen (31–33). Moreover, γδ T cells are also widespread within epithelial-rich tissues that form the inner and outer surfaces of the body, such as the reproductive tract, intestinal epithelial cells, and skin epidermis and dermis (34–38). They have the potential to be the source of γδ T cells infiltrating into the ischemic brain, which will be mentioned later.

Strikingly, a unique feature of murine γδ T cells is the preferential expression of different Vγ segments in different tissues. For example, Vγ5 + γδ T cells are present in the epidermis, Vγ7 + γδ T cells lie in the gut epithelia, Vγ6 + γδ T cells localize to the reproductive mucosa (39). Likewise, in humans, Vδ1 + T cells are a major subset in many tissues, such as thymus, spleen (40), breast (41), decidua (42), liver (43), lung, intestinal epithelia (44, 45) and skin dermis and epidermis (46), and Vγ9Vδ2 T cells are the major γδ T cell subset in the peripheral blood. Understanding of γδ T cell subtypes and their specific distribution fully and then developing therapies that target the specific cell subtype and their tissue specificity could increase effectiveness and reduce side effects of medicines or other treatments.

## The Role of γδ T Cells in Cerebral Ischemic Stroke

### Source of γδ T Cells

In the context of stroke, γδ T cells infiltrate into affected brain parenchyma and leptomeninges and participate in the inflammatory and immune responses in brain ischemic injury (39, 47). CC chemokine receptor 6 (CCR6) is required for the infiltration of IL-17-producing γδ T cells in experimental stroke. Genetic deficiency of CCR6 is associated with diminished infiltration of IL-17-producing γδ T cells and a significantly improved neurological outcome (48). In addition, dysbiosis in some tissues also influences the infiltration of γδ T cells. The article by Benakis et al. (47) made the point that intestinal dysbiosis altered immune homeostasis in the small intestine, d decreased the number of γδ T cells in the meninges, suppressed the function of effector IL-17-positive γδ T cells, and then affected ischemic stroke outcome. Inspired by the proximity, Brea et al. explored and found that nasal-associated lymphoid tissue (NALT) is not the source of stroke-associated IL-17a + γδ T cells (49). Whether γδ T cells can migrate from tissues with a high frequency of γδ T cells mentioned above, such as reproductive tract and skin dermis, remains to be further explored and confirmed. Moreover, the specific mechanisms leading to the migration of γδ T cells remain to be further elucidated. Recently, de Lima et al. (34) have found a high representation of γδ T cell receptor-expressing cells without expression of the conventional T cell coreceptors CD4 and CD8 in the dura mater. Of note, these cells can regulate anxiety-like behavior via IL-17a signaling. It is worth considering whether dura-associated γδ T cells are involved in the pathophysiology of CIS just like infiltrated γδ T cells; certainly, if they do, the mechanisms they work in CIS need to be explored.

### Activation of γδ T Cells

γδ T cells express IL-23 receptor (IL-23R), IL-18R (50, 51). The receptors are combined with IL-23, IL-1β, and IL-18, which are produced from dendritic cells and infiltrated macrophages, rather than residential microglia, and then γδ T cells are stimulated to produce IL-17a, IL-21, IL-22, and IFN-γ, in the absence of TCR engagement (51–53). In addition, γδ T cells express Toll-like receptor (TLR) 7, TLR9, and the central TLR adapter molecular MyD88 intracellularly, and express TLR2 on the cell surface. However, upon TLR stimulation with TLR ligands, γδ T cells do not secret IL-17a (54). McCandless et al. (55) have reported that γδ T cells can secrete IL-1β in experimental autoimmune encephalomyelitis (EAE), and depletion of γδ T cells can decrease IL-1β levels. However, whether γδ T cells can secrete IL-1β in CIS has not been demonstrated.

Most of these molecules mentioned above are involved in the pathological process of ischemic brain injury. Besides, in the fields of anti-bacterial immunity and tumor immunity, γδ T cells can be activated by phosphoantigens (pAgs), which includes (E)-1-hydroxy-2-methyl-but-2enyl pyrophosphate (HMBPP), dimethylallyl pyrophosphate (DMAPP) and isopentyl pyrophosphate (IPP), through γδ TCR (56, 57). And butyrophilins (BTN)/butyrophilin-like molecules (BTNL) are core mediators of pAg sensing by γδ T cells (58). What's more, little research has focused on the signaling pathways that exist in γδ T cells intracellularly after activation through ligands combined with their receptors. The small amount, the difficulty to extract, and no cell line are characteristics of γδ T cells and may be why they are short of research. Noteworthy, these signaling pathways are worth studying to understand the pathogenic process in CIS more sufficiently and to develop more effective therapies.

### Potential Mechanisms of the Effect of γδ T Cell-Derived Cytokines on CIS

**Figure 1:**
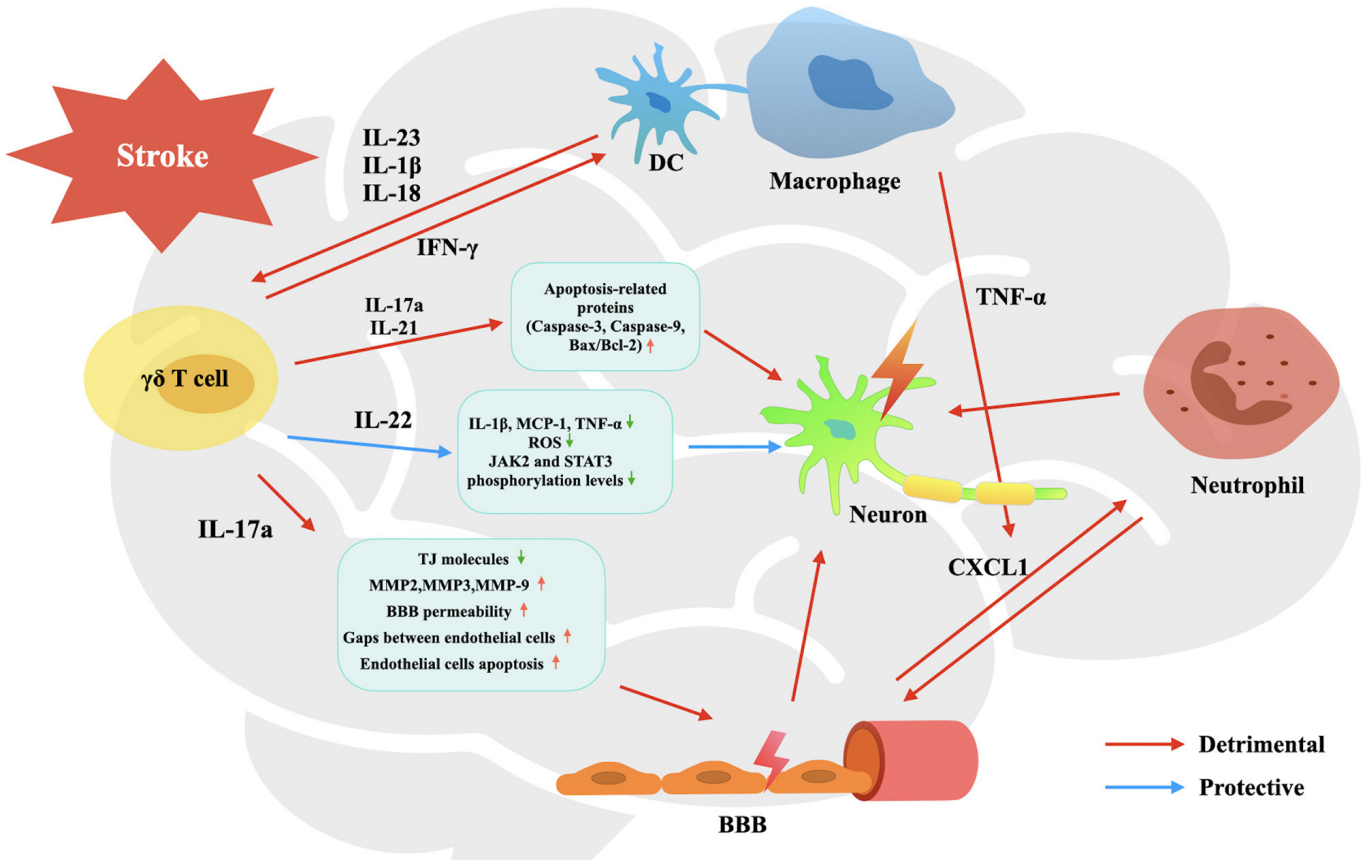
Activation and effects of γδ T cells in the pathophysiology process of CIS. Upon activated by IL-23, IL-1β and IL-18, infiltrated γδ T cells are stimulated to produce IL-17a, IL-21, IL-22, and IFN-γ. They participate in the pathophysiological process of CIS, involving promoting BBB breakdown, neutrophil infiltration, neuronal cell apoptosis and autophagy, and so on, leading to affected brain tissue damage and neurological deficit.

γδ T cells exert an important effect on brain tissue injury in CIS not by themselves, but mainly by cytokines they release, including IL-17a, IL-21, IL-22, and IFN-γ. Each of them plays a unique role in the process of cerebral ischemia and reperfusion injury (Figure 1), including promoting BBB breakdown, neutrophil infiltration, neuronal cell apoptosis and autophagy, and so on, resulting in unreversible brain damage.

#### IL-17a

It has been reported that in both experimental models and patients, IL-17a is augmented in brain tissue and peripheral blood after CIS. γδ T cells are the main sources of IL-17a in the context of stroke (52). Other innate and adaptive immune cells, including Th17 cells, natural killer T (NKT) cells, natural killer (NK) cells, group 3 innate lymphoid cells (ILC3s), and neutrophils have been found to secrete IL-17a as well (59, 60). As previously reported, IL-17a-releasing γδ T cells peak on day 3 after the onset of ischemia, and IL-17a seems to have a crucial role in the maturation of brain infarction (59).

Firstly, IL-17a could promote neutrophil infiltration. By binding with IL-17R, which is expressed on glial cells and brain microvascular endothelial cells (BMECs) and is up-regulated after stroke in experimental models, IL-17a promotes glia and BMECs to secret and activate large amounts of CXCL1; CXCL1 is a neutrophil chemoattractant that could lead neutrophils to infiltrate into the affected cerebral parenchyma (61–65). Moreover, augmented infiltrating neutrophils can further destroy the integrity of the blood-brain barrier (BBB), and promote the lysis and apoptosis of nerve cells, as a result, to aggravate brain injury (18, 66, 67).

Secondly, IL-17a could destroy the structural integrity of BBB. Huppert et al. (68) have found that IL-17a could induce ROS production so that to down-regulate the expression of tight junction (TJ) molecule occluding. Ni et al. (69) have verified that IL-17a could elevate matrix metalloproteinase (MMP)-2, MMP-3, and MMP-9 in BMECs to hydrolyze TJs, leading to increased BBB permeability. Zhu et al. (70) have uncovered that IL-17a induced the production of von Willebrand factor by endothelial cells, and promoted endothelial cells apoptosis by activating caspase-3 and caspase-9 and up-regulating the ratio of Bcl-2-associated X protein (Bax)/B cell lymphoma/leukemia-2 (Bcl-2). However, the aforementioned damaging effect of IL-17a on BBB has only been confirmed in other models of brain inflammatory diseases, and the effect needs to be verified in CIS.

Thirdly, IL-17a promotes neuron death, including apoptosis and autophagy. IL-17a facilitates the expression of apoptosis-related proteins including caspase-3, caspase-9, and Bax, and increases the ratio of Bax/Bcl-2 after brain injury, thus resulting in neuron apoptosis (71). Liu et al. (72) have also found that IL-17a could mediate excessive autophagy to aggravate neuronal ischemic injuries via the Src-PP2B-mTOR pathway.

All the damaging effects above lead to larger infarction and worse outcomes. Likewise, IL-17a and γδ T cells have also been demonstrated to be implicated in human stroke. Infiltration of γδ T cells and secretion of IL-17a have been documented in ischemic human brain tissue and circulating IL-17a is elevated as well (53, 61, 73). Later, clinical research is needed to verify the role of the γδ T cell-IL-17a axis in patients suffering from ischemic stroke and potential mechanisms, so that to develop more effective therapies applied to clinical practice.

Noteworthy, IL-17a plays a dual role at different time points after ischemic stroke in mice. The article by Lin et al. makes the point that IL-17a showed two apparent peaks of expression in the ischemic hemisphere: one occurring within 3 days, which is secreted from γδ T cells and has detrimental roles in the pathogenesis of acute ischemic stroke as mentioned above, and the other on 28 d after stroke. And astrocytes are the major cellular source of the second peak of IL-17a that has a property in the maintenance and augment of survival and neuronal differentiation of subventricular zone (SVZ) neural precursor cells (NPCs), and subsequent synaptogenesis and functional recovery after ischemic stroke (74). Whether promoting IL-17a releasing or exogenous administration of IL-17a during convalescence improves stroke outcomes remains to be explored and verified.

#### IL-21

In a mouse model of transient middle cerebral artery occlusion (tMCAO), IL-21 is robust up-regulated in the injured brain, and IL-21 exerts a pronounced effect on brain injury via up-regulating autophagy-related genes of neuronal cells which express IL-21R. In addition, in postmortem human brain tissue, IL-21 was also found in the area surrounding acute stroke lesions, suggesting that IL-21-mediated brain injury may be relevant to human stroke (75). IL-21 gene owns two polymorphisms, rs907715G/A and rs4833837A/G, and the former causes the augment of IL-21 mRNA and protein levels in peripheral blood mononuclear cells (PBMCs) and shows a positive correlation with brain ischemic injury; thus, IL-21 may be important in the development of the disease (76). Therefore, there is a potential for regulation at the protein level, even genetic level, to prevent and mitigate ischemic stroke in the brain.

Surprisingly, Weiner et al. (77) and Lee et al. (78) found that IL-21R exerted a neuroprotective effect via Janus tyrosine kinase (JAK)/signal transducer and activator of transcription (STAT) signaling pathways and upregulation of caspase-3, and neuronal cell death and infarct volume increased in IL-21R-deficient mice suffered from ischemia compared with the control group. Given that the protective effect of IL-21R was more evident in permanent middle cerebral artery occlusion (pMCAO) than in tMCAO, and T lymphocytes infiltration, which was the source of IL-21, was obvious in tMCAO while poor in pMCAO, the reason for the difference above may be the source of IL-21 and IL-21R, resident or infiltration, and maybe also related to a collateral-independent effect on cerebral injury (78). Therefore, the exact role of IL-21 in CIS requires more evidence to elucidate.

#### IL-22

IL-22 exerts a protective effect against CIS. Dong et al. have found that injecting intraperitoneally with recombinant mouse IL-22 protein (rIL-22) into mice could reduce the expression of inflammatory cytokines, including IL-1β, monocyte chemotactic protein (MCP)-1 and tumor necrosis factor (TNF)-α, both in the serum and the ischemic cerebral cortex. In addition, IL-22 treatment also decreased oxidative stress and neuronal apoptosis in affected brain tissue. Moreover, treatment with IL-22 significantly increased JAK2 and STAT3 phosphorylation levels in mice and PC12 cells. The effects mentioned above lead to the reduction of infarct size, neurological deficits, and brain water content in mice subjected to CIS (79). There is a problem that IL-22 is administered exogenously, but not γδ T cell-derived. The research about the role of γδ T cell-derived IL-22 in brain ischemic stroke is rare, and the neuroprotective effect of IL-22 in stroke needs more evidence to verify.

#### IFN-γ

In mice suffering from stroke, Th1 cells are the main source of IFN-γ, and a very few γδ T cells could produce IFN-γ (52). Similarly, using a fate-tracking system, Hirota et al. (80) have found that <20% of γδ T cells were shown to express IFN-γ in the central nervous system (CNS) in the context of EAE. The same as the IL-17a, IFN-γ-producing cells strongly accumulate by day 3 after ischemia in affected brain tissue and decrease thereafter (52). Certainly, IFN-γ participates in the pathophysiological process of CNS-related diseases. In some central nervous systems (CNS)-related diseases, for example, spinal cord injury (SCI), γδ T cells are detected at the lesion sites and express the inflammatory cytokine IFN-γ, which induces macrophages to transform into M1 phenotype with increased secretion of proinflammatory cytokines, such as TNF-α (81). Likewise, the article by Gelderblom et al. makes the point that IFN-γ produced by CD4+ T cells could induce TNF-α production from macrophages. And synergistic with TNF-α, IL-17a could enhance astrocytes to secrete CXCL1, leading to enhanced neutrophil infiltration in a mouse model of stroke (61). Blocking IFN-γ or inactivating Vγ4+ γδ T cells with antibodies has a beneficial effect and improves the functional recovery after SCI (81). Therefore, manipulation of γδ T cell and IFN-γ functions may be a promising approach for CIS treatment in the future.

#### IL-1β

On one hand, through combination with IL-18R expressed on γδ T cells, IL-1β are involved to stimulate IL-17a production by γδ T cells (51). On the other hand, IL-1β may be secreted by γδ T cells (55). As reported by Mccandless et al., in the rodent model of multiple sclerosis (MS), EAE, the inflammatory cytokine IL-1β, γδ T cells are one of the sources of which, participates in the pathogenic process through mediating pathologic relocation of CXCL12 and disruption of BBB (55). In addition, IL-1β is involved in perpetuating immune responses and contributing to disease severity in a variety of CNS diseases, such as neurodegenerative diseases, traumatic brain injury, and diabetic retinopathy (82). Moreover, it is beneficial to block IL-1β signaling in some autoimmune and autoinflammatory diseases, making IL-1β a potential therapeutic target in neuroinflammatory conditions, including CIS. However, the evidence that γδ T cells secrete IL-1β is particularly rare, and more research is needed to support this.

These five cytokines mentioned above that play a role in brain stroke are released partly from T lymphocytes, but not specifically from γδ T cells, thus, there remains a lot of research to be done to confirm the role of the specific γδ T cell-derived cytokines in cerebral ischemia and reperfusion injury to further improve the mechanisms.

## Therapies for Stroke Targeting at γδ T Cells

γδ T cells and related cytokines have essential roles in ischemic brain injury. Treatments targeting γδ T cells and their cytokines could be good therapeutic targets for mitigating ischemic brain damage, given the truth that either genetic disruption or pharmacological blockade of γδ T cells, IL-17a or IL-21 shows a significant neuroprotective effect on ischemic brain damage in murine stroke (Table 1). Of note, in other neuroinflammatory diseases, blockade of IFN-γ and IL-1β signaling also have a neuroprotective effect, while their beneficial effect has not been verified in CIS. In addition, through modifying the composition of the gut microbiota, Benakis et al. found that intestinal dysbiosis could reduce ischemic brain injury. The mechanism was shown to be related to a reduction of IL-17-positive γδ T cells and an increase of regulatory T (Treg) cells through altering dendritic cell activity. And dysbiosis could suppress the trafficking of effector T cells from the gut to the leptomeninges after stroke. The findings uncovered a gut-brain axis, which was unrecognized previously, and an impact of the intestinal flora and meningeal IL-17+ γδ T cells on ischemic brain damage (47). Inspired by this article, there remains a possibility that diseases with altering immune homeostases, such as inflammatory bowel disease (IBD), Crohn's disease (CD), and systemic lupus erythematosus (SLE), could affect ischemic stroke outcomes by impacting the trafficking of γδ T cells into ischemic brain tissue.

**Table 1:** Therapies targeting at γδ T cells and related cytokines in CIS.

**Type**	**Gender**	**Age**	**Weight**	**Model**	**Intervention**	**Potential Mechanisms**	**Effect on stroke**	**References**
C57BL/6 mice	-	12 weeks	20–25 g	tMCAO	Mouse monoclonal anti–murine IL-17A antibody (Clone MM17F3) (i.p.)	Reduce neutrophil infiltration	Protective	(61)
C57BL/6 mice	Male	9–17 weeks	20–30 g	tMCAO	FTY720 (i.v.)	Inhibit T lymphocytes, including γδ T cells, migration into inflammatory tissues	Protective	(52)
					IL-17KO mice	Reduce mRNA expression levels of inflammatory factors, including IL-1β, TNF-α and MMPs; reduce apoptotic neurons	Protective	
					TCRγδ-deficient (TCRγδ KO) mice	Reduce mRNA expression levels of inflammatory factors, including IL-1β, TNF-α	Protective	
					TCRγδ-specific antibody (i.p.)			
SD rats	Male	-	250–300 g	tMCAO	Cholera toxin B subunit (CTB) (i.p.)	Reduce the levels of γδ T cells, IL-17-producing γδ T cells, and IL-17	Protective	(83)
C57BL/6 mice	Male	7 weeks	≥20 g	tMCAO	Alter the intestinal flora by antibiotic	Suppress function of effector IL-17+ γδ T cells and trafficking of γδ T cells from the gut to the leptomeninges	Protective	(47)
C57BL/6 mice	Male	8–10 weeks	23–25 g	tMCAO	Anti-IL-17A monoclonal antibody treatment (i.v.)	Decrease calpain-mediated alpain-transient receptor potential canonical (TRPC)6 channel degradation	Protective	(84)
					IL-17A knockout			
C57BL/6 mice	Male	8–10 weeks	25 g	tMCAO	CP-690550 (i.p.)	Suppress IL-17 production from T cells	Protective	(85)
					Anti-p40 antibody (i.p.)	Suppress the infiltration of IL-17-positive γδT cells	Protective	
SD rats	Male	-	270–320 g	pMCAO	Bone marrow mesenchymal stem cells (BMSCs) (i.v.)	Reduce the infiltration of γδ T cells and increase Tregs.	Protective	(86)
C57BL/6 mice	-	-	-	tMCAO	miR-215 mimic (i.c.v.).	Suppresses autophagy by inhibiting the Act1/IL-17RA and JNK/pBcl-2/Beclin pathways	Protective	(62)
C57BL/6 mice	Male	7–10 weeks	21–30 g	tMCAO	1α, 25-dihydroxyvitamin D3 (1,25-VitD3) (i.p.)	Reduce the expression of pro-inflammatory mediators IL-6, IL-1β, IL-23a, TGF-β, NADPH oxidase-2 and ROR-γt, and reduce Th17/γδ T cell response	Protective	(87)
C57BL/6 mice	Male	-	-	tMCAO	Mouse monoclonal anti–murine IL-17A antibody (Clone MM17F3) (i.v.)	Decrease neutrophil levels	Protective	(48)
					CCR6^−/−^ mice	Reduce the infiltration of IL-17+ γδ T cells and neutrophil	Protective	
C57BL/6 mice	Male	12 weeks	20–25 g	tMCAO	Depletion of CD11c+ cells or the genetic disruption of the IL-23	Abrogate both IL-17 production in γδ T cells and neutrophil infiltration	Protective	(88)
C57BL/6 mice	Male	8–12 weeks	-	pMCAO	Perforin 1^−/−^ mice	Reduce the number of γδ T cells and IL-17 levels	Protective	(89)
C57BL/6 mice	Male	8–12 weeks	20–30 g	tMCAO	Recombinant murine IFNβ (i.v.)	Reduce the infiltration of monocytes/macrophages, neutrophils, CD4+ T cells, and γδ T cells; inhibits the production of inflammatory mediators; suppress the expression of adhesion molecules on brain endothelial cells; repress microglia activation in the ischemic brain	Protective	(90)
C57BL/6 mice	-	-	25 g	tMCAO	IL-21 receptor Fc protein (IL-21R.Fc) (i.p.)	Decrease mRNA levels of the autophagy associated gene ATG6	Protective	(75)
					IL-21–deficient mice (IL-21^tm1Lex^)			
C57BL/6 mice	Male	-	-	tMCAO	Recombinant mouse IL-22 protein (rIL-22) (i.p.)	Decrease oxidative stress and neuronal apoptosis and increase JAK2 and STAT3 phosphorylation levels	Protective	(79)

Nevertheless, in human stroke, the evidence that treatments targeting γδ T cells and cytokines they release are beneficial to the outcomes is rare. Caccamo et al. (91) have found that in the peripheral blood and at the site of disease in children with bacterial meningitis, the percentage of IL-17+Vγ9Vδ2 T lymphocytes was increased, while this pattern was reversed after successful antibacterial therapy. This article indicated that IL-17+γδ T cells participated in the neurological disease, and γδ T cells might be the target of the antibiotic therapy. Moreover, blockade of the proinflammatory cytokine IL-17a with secukinumab, a fully human selective anti-IL-17a monoclonal antibody, is a valid therapeutic approach and may be useful in the treatment of psoriasis, Rheumatoid Arthritis (RA), and noninfectious uveitis (92). Whereupon, γδ T cells and cytokines they release are therapeutic targets with great potential and are worth studying.

However, there is controversy over the efficacy of therapies targeting γδ T cells and related cytokines. Adamski et al. (93) did not find associations of γδ T cell counts with lesion volume, stroke severity, and outcome in the clinical study. However, it does not mean that γδ T cells do not exert effects on human stroke. And a large patient sample is needed to verify their associations. Meanwhile, several anti-IL-17a drugs are in clinical trials for some inflammatory disorders, such as IBD and CD. Targeted inhibition of IL-17a by secukinumab is ineffective in patients with moderate to severe CD and adverse events are noted compared with placebo (94). A separate line of evidence also reports that IL-17a acts on intestinal epithelium to promote barrier function, whereas IL-17a or IL-17 receptor A (IL-17RA) inhibition promotes severe weakening of the barrier, culminating in increased colonic inflammation and accelerated mortality (95). Similar results obtained report that IL-17 regulates occludin protein that limits gut excessive permeability and maintains barrier integrity during an epithelial injury in a dextran sodium sulfate (DSS) model of IBD, while neutralizing IL-17 causes increased gut permeability (96), further confirming the deleterious effects of neutralizing IL-17a and IL-17RA.

The dichotomy in the efficacy of therapies targeting γδ T cells and related cytokines does exist. Since then, there is a long way to go to verify that γδ T cells and related cytokines are promising therapeutic targets in patients attacked by CIS.

## Concluding Remarks

CIS is an increasing threat to endanger health and safety all over the world. A better understanding of relations between brain tissue damage after ischemic stroke and inflammatory and immune responses remains to be improved to shed light on the development of more effective therapies in the future. γδ T cells, a small subset of T cells, regulate the inflammation process in many diseases, including CIS. After the onset of ischemic stroke, upon activation, γδ T cells can release IL-17a, IL-21, IL-22, and IFN-γ, and then participated in the pathogenic process, including secretion of pro-inflammatory factors, breakdown of the integrity of BBB, and recruitment of inflammatory cells into the affected tissues, and eventually cause irreversible brain injury. A variety of researches have demonstrated that suppressing the infiltration of γδ T cells and reduction of IL-17a and IL-21 levels via either pharmacological or genetic tools could improve the outcome of stroke. Noteworthy, our experiments aim to benefit patients attacked by stroke, hence more evidence is required to confirm whether Pre-clinical pathogenic mechanisms mentioned above are also appropriate to patients suffering from CIS. This is to provide a more sufficient basis for clinical application. In conclusion, γδ T cells and related cytokines play a vital role in the stroke and may be therapeutic targets with great potential for treatment.

## Author Contributions

LW and CY contributed to editing the manuscript and discussed the topic and structure of this article together. SY and YL provided administrative support. JC, YG, CW, YW, FW, YS, and MD helped with the manuscript editing and discussions. All authors contributed to the review and approved the submitted version.

## Funding

This work was supported by the Major Technological Innovation Special Project of Hubei Province of China (2019ACA167) 2019 College-level Teaching Reform Research Project (02.03.2019.15-15).

## Conflict of Interest

The authors declare that the research was conducted in the absence of any commercial or financial relationships that could be construed as a potential conflict of interest.

## Publisher's Note

All claims expressed in this article are solely those of the authors and do not necessarily represent those of their affiliated organizations, or those of the publisher, the editors and the reviewers. Any product that may be evaluated in this article, or claim that may be made by its manufacturer, is not guaranteed or endorsed by the publisher.
